# Randomised controlled feasibility trial of a web-based weight management intervention with nurse support for obese patients in primary care

**DOI:** 10.1186/1479-5868-11-67

**Published:** 2014-05-21

**Authors:** Lucy Yardley, Lisa J Ware, Emily R Smith, Sarah Williams, Katherine J Bradbury, Emily J Arden-Close, Mark A Mullee, Michael V Moore, Janet L Peacock, Mike EJ Lean, Barrie M Margetts, Chris D Byrne, Richard FD Hobbs, Paul Little

**Affiliations:** 1 Centre for Applications of Health Psychology (CAHP), Faculty of Social and Human Sciences, University of Southampton, Southampton, UK; 2 Hypertension in Africa Research Team (HART), North-West University, Mahikeng, South Africa; 3 Psychology Research Centre, Faculty of Science and Technology, Bournemouth University, Bournemouth, UK; 4 NIHR Research Design Service South Central, Faculty of Medicine, University of Southampton, Southampton, UK; 5 Primary Care and Population Sciences, Faculty of Medicine, University of Southampton, Southampton, UK; 6 Division of Health and Social Care Research, King’s College London; NIHR Biomedical Research Centre at Guy’s and St Thomas’ NHS Foundation Trust and King's College London, London, UK; 7 Human Nutrition, School of Medicine, University of Glasgow, Glasgow, UK; 8 Southampton National Institute for Health Research (NIHR) Biomedical Research Centre, University Hospital Southampton and University of Southampton, Southampton, UK; 9 Nuffield Department of Primary Care Health Sciences, Faculty of Medicine, University of Oxford, NIHR SPCR, NIHR Oxford BRC, and NIHR Oxford CLAHRC, Oxford, UK

**Keywords:** Weight reduction programme, Internet, Randomized controlled trial, Obesity, Nurse support

## Abstract

**Background:**

There is a need for cost-effective weight management interventions that primary care can deliver to reduce the morbidity caused by obesity. Automated web-based interventions might provide a solution, but evidence suggests that they may be ineffective without additional human support. The main aim of this study was to carry out a feasibility trial of a web-based weight management intervention in primary care, comparing different levels of nurse support, to determine the optimal combination of web-based and personal support to be tested in a full trial.

**Methods:**

This was an individually randomised four arm parallel non-blinded trial, recruiting obese patients in primary care. Following online registration, patients were randomly allocated by the automated intervention to either usual care, the web-based intervention only, or the web-based intervention with either basic nurse support (3 sessions in 3 months) or regular nurse support (7 sessions in 6 months). The main outcome measure (intended as the primary outcome for the main trial) was weight loss in kg at 12 months. As this was a feasibility trial no statistical analyses were carried out, but we present means, confidence intervals and effect sizes for weight loss in each group, uptake and retention, and completion of intervention components and outcome measures.

**Results:**

All randomised patients were included in the weight loss analyses (using Last Observation Carried Forward). At 12 months mean weight loss was: usual care group (n = 43) 2.44 kg; web-based only group (n = 45) 2.30 kg; basic nurse support group (n = 44) 4.31 kg; regular nurse support group (n = 47) 2.50 kg. Intervention effect sizes compared with usual care were: d = 0.01 web-based; d = 0.34 basic nurse support; d = 0.02 regular nurse support. Two practices deviated from protocol by providing considerable weight management support to their usual care patients.

**Conclusions:**

This study demonstrated the feasibility of delivering a web-based weight management intervention supported by practice nurses in primary care, and suggests that the combination of the web-based intervention with basic nurse support could provide an effective solution to weight management support in a primary care context.

**Trial registration:**

Current Controlled Trials ISRCTN31685626.

## Background

Obesity is a major threat to public health internationally, and the prevalence has risen sharply in recent decades [1,2]. Meta-analyses suggest that behavioural weight management interventions, which help people to monitor and regulate their relative dietary intake and energy expenditure, on average result in clinically useful weight loss [3,4]. Given the high levels of morbidity associated with obesity, there is a demand for health professionals in primary care to provide weight management interventions for their obese patients. The problem is that most practice staff have neither the training nor the time to implement intensive in-person counselling for weight management for the large numbers of obese patients who would like such support [5]. Automated web-based programmes to support weight management might offer a potential solution [5,6]. However, there is huge variability in the outcomes of web-based weight management programmes, with a trend towards better outcomes in programmes with additional personal support [7,8]. Given the high cost of intensive, personal support, it is vital to try to determine the optimal combination of web-based and personal support [7].

Some studies of fully automated web-based interventions with no human contact have reported similar weight loss in intervention and control groups, suggesting that some human support may be crucial [9,10]. A number of studies have provided direct comparisons between various levels and formats of support. Face-to-face support typically results in better outcomes than purely internet-based support for both weight loss and weight maintenance [11,12]. However, studies of providing intensive online in-person counselling to users of an automated website have found that this can also significantly improve outcomes - but note that intensive online counselling is nearly as costly as face-to-face support, thus reducing the cost-effectiveness of the intervention [13,14]. When a web-based intervention was supplemented by weekly phone calls for 3 months, followed by monthly phone calls throughout the 24 month study, adding face-to-face group sessions produced very little extra benefit [15].

It is difficult to extrapolate from most of these studies directly to provision of web-based weight management support in the UK primary care context, as many factors can affect the requirements for online provision and human support. For example, one study that did achieve better outcomes than the control group with only automated support noted that their volunteer sample was highly educated and motivated [13], and so may have needed less support than typical primary care patients. The only UK study of Internet-based weight management in a (university) primary care setting observed slightly greater weight loss in the control group, and concluded that this was because no human support was provided [10]. The main aim of the study reported here was therefore to build on previous experiences of providing nurse-led weight management in a UK primary care context [16] to carry out a feasibility trial of providing a fully automated web-based weight management intervention in primary care, comparing different levels of nurse support. The key requirements for primary care are that interventions must result in sustainable weight loss [17] and be feasible and cost-effective to deliver. This feasibility trial was carried out as preparation for a fully-powered trial to determine the cost-effectiveness of the web-based intervention compared with usual care, with provision of whatever nurse support seemed likely from the feasibility trial to prove most cost-effective. Our main objective in this feasibility trial was therefore to examine the longer-term (12 month) weight loss outcomes that could be achieved with less intensive levels of support than those employed in previous successful trials of weight management. As this was a feasibility trial [18], the variables we examined included dose/efficacy effects of the intervention with different levels of nurse support, levels of adherence to the intervention, and the feasibility of our trial processes. To increase patient motivation we offered a choice of low calorie and low carbohydrate eating plans; since there have been few direct comparisons of these alternative approaches to weight loss [19], we were also interested in patient preferences and outcomes relating to each eating plan.

## Methods

### Design

The design was a randomised feasibility trial comparing four parallel groups: usual care; web-based only; website with basic nurse support; website with regular nurse support. Before commencing, the trial was approved by the UK National Health Service (NHS) National Research Ethics Service and was registered with Current Controlled Trials (number ISRCTN 31685626). As this was a feasibility trial to inform the design of a subsequent full trial, our target sample size was around 50 patients per group (which is normal for a dose response study of this kind [20]), which would allow us to estimate: the likely relationship between level of nurse support and change outcome; the variability and the variability of change in outcomes in this population; and any further issues of feasibility or acceptability of the intervention and/or trial procedures.

### Participants and procedures

Patients were recruited between May 2011 to December 2012 from five general practices in southern England. Adult patients (aged at least 18) were included if they had a BMI > =30 (or 28 with hypertension, hypercholesterolaemia or diabetes) documented in the medical records; only one patient per household could participate. Patients were excluded if they were pregnant or breast-feeding, had current major mental or physical health problems (i.e. unable to change their diet or complete trial procedures) or had self-reported inability to walk 100 metres (i.e. physical activity difficult).

Most patients were recruited by a letter of invitation from the practice enclosing the Patient Information Sheet, but primary care staff could personally hand out the study details to suitable patients and a poster in the practice waiting rooms invited patients to ask for study details. Interested patients met the practice nurse for a discussion of the study and confirmation of eligibility, and to give informed consent. At this meeting consenting patients then received their baseline nurse assessment and were given a unique study ID to login to the website. When patients first logged into the website (in their own homes) they completed baseline self-report measures of attitudes and behaviour. Participants were then automatically randomised to one of the four groups by a computer algorithm that employed stratification by waist (allocating to the lower weight group if waist < 88 cm for women, < 102 cm for men), and a block size of 60 within each practice. The computer system immediately informed participants which group they had been allocated to, and sent an email to inform the practice nurse. For this feasibility study it was not subsequently possible to conceal treatment allocation from participants, nurses performing follow-up, or the research team members providing support to users and nurses.

Follow-up nurse assessments were carried out at six months and one year. At six months practice nurses sent patients an appointment to attend the practice for the full set of physiological measures, including weighing and a fasting blood sample, and practice staff followed up non-respondents by telephone. As this procedure appeared to be contributing to dropout, ethical approval was obtained for different follow-up procedures at twelve months. At the final follow-up patients were sent a standard letter encouraging them to contact the practice to arrange the appointment, enclosing a £10 voucher and giving the patient the option of not having a fasted blood sample taken. Non-respondents were then sent a second letter offering them the option of being weighed in their own home by a member of the research team. Non-respondents to this letter then received a telephone call from the research team to schedule an appointment at home, or if a follow up could not be scheduled to offer the patient the opportunity to self-report their current weight. Self-report measures of attitudes and behaviour were re-administered automatically by the website at six months and one year, with two email reminders to non-respondents; patients who did not complete the measures online were sent a paper version to complete and return by post in a pre-paid envelope. Self-report measures of views of the intervention were completed only by the patients in the intervention groups.

### Intervention

The web-based intervention (Positive Online Weight Reduction; POWeR) was designed to provide support for self-management of weight based on patient choice of either a low calorie or a low carbohydrate eating plan. The low calorie eating plan suggested a reduction of around 600 calories a day. A reduction in portion size of 25% for women and 20% for men was suggested as a simple way of achieving this. Alternatively, users could base their eating plan on a traffic light system that categorised foods as those that could be eaten freely (‘green’), in moderation (‘orange’) or only in very small quantities (‘red’). The low carbohydrate eating plan allowed a carbohydrate limit of 50 g a day, and also used a traffic light system to categorise foods, based on their carbohydrate content. On the advice of the nutrition experts on our team, this system categorised most vegetables and some fruits as ‘green’ and only categorised very high sugar and starchy foods as ‘red’ and was therefore compatible with a sustainable healthy diet. Patients were also encouraged to increase their physical activity levels by choosing either a walking plan (in which case a pedometer was supplied [21]) or a self-selected mixture of other physical activities.

The intervention was intended to foster self-regulation skills to enable users to autonomously self-manage their weight [22], and drew on cognitive-behavioural techniques [23] to address problems such as motivation and relapse. Because our objective was long-term weight loss maintenance we wished to minimise the cognitive burden for users, and so the emphasis was on forming sustainable healthy eating and physical activity habits rather than daily calorie counting, using weekly weight monitoring to check the effectiveness of chosen eating and physical activity goals for managing weight. Daily food and activity records (and calorie counting if necessary) were recommended as short-term diagnostic tools to use initially or if not losing weight. Nevertheless, to ensure that sufficiently demanding goals were set, the first session involved completing a retrospective food diary for the past 24 hours to identify high calorie/carbohydrate foods to omit or substitute. The web-based goal-setting tool also required patients to choose their first weekly goal from pre-set choices likely to promote significant weight loss (e.g. avoid all ‘red’ foods, eat ‘amber foods’ only once a day, reduce main meal portion sizes by 25%, avoid all high calorie/carbohydrate snacks or drinks), and recommended choosing a second goal from this list (in addition to creating their own personal goal). The intervention was developed based on behavioural theory and evidence from existing successful interventions [3,16,24], with extensive iterative qualitative piloting to check usability, accessibility and acceptability and to elicit and respond to user views. Further details of the intervention development are given elsewhere (including a complete taxonomy of behaviour change techniques used) [25].

The format of the POWeR intervention is a set of twelve weekly sessions. Throughout POWeR, users are taught active cognitive and behavioural self-regulation techniques (‘POWeR tools’) with evidence for their effectiveness and examples of how others have successfully used them (‘POWeR stories’). Session 1 provides an overview of the POWeR intervention and advice on choosing low calorie or low carbohydrate eating plan, helps users to set eating goals and plan how to implement them, asks users to identify their personal reasons for losing weight, and explains how to use weekly weighing for self-monitoring. All subsequent sessions begin by asking the user to enter their current weight and report how often they have achieved each of the goals set the previous week; users then receive advice based on progress (e.g. positive feedback if successful, advice on overcoming barriers if unsuccessful). Users can then access new content each week (as well as a graph of their progress and their ‘POWeR tools’). Session 2 covers getting support from the website (e.g. setting automated motivational messages), friends and family, and the nurse. Session 3 helps users choose and implement a physical activity plan. After completing the first 3 sessions users can choose weekly sessions covering: cravings; relapse; increasing physical activity; emotional eating; eating when busy; environment restructuring; alcoholic and non-alcoholic drinks; eating out. The final session focuses on weight maintenance. All sessions are tailored to gender and finish with a set of recommended links to other relevant high quality websites.

### Intervention arms

The *web-based only* group received no scheduled support and no face to face or telephone contact with the nurse between six monthly assessments, although all patients were permitted to email the nurse via the website if they had a specific query. The group with *basic nurse support* had scheduled support two weeks, one month and three months after baseline (a total of three contacts scheduled). The group with *regular nurse support* had scheduled support at two weeks and then monthly for the first six months of the study (a total of seven contacts). Support was offered as a 15–20 minute face-to-face session, or by telephone or email if the patient could not attend a face-to-face meeting. Nurse support was guided by brief structured training materials accessed on the intervention website, which asked them simply to provide positive reinforcement and encourage patients to find their own solutions for weight management with the help of the website (rather than rely on the nurses for advice). Nurses could log onto the intervention website before support sessions to check patient logins, weekly weight entries and current goals. In the *usual care* group, access to interventions and support was determined by the primary care staff; this group was also offered access to the website at the end of the study.

### Measures

The main outcome measure (intended as the primary outcome for the main trial) was weight in kg at twelve months (assessed lightly clothed, without shoes, using clinically validated and calibrated scales; Tanita, Japan). The 12 month follow-up was selected as the primary outcome because the aim of the programme was to promote sustained weight management, and 12 months is typically regarded as an indicator of maintained weight loss [17]. For this feasibility trial, it was also important to establish uptake and dropout rates and levels of completion of the primary and secondary outcomes. Since weekly weighing and goal-setting (which triggered tailored progress-related advice) was the core of the POWeR intervention and was compulsory on logging on, adherence to the website intervention by patients was assessed quantitatively by number of weekly weight and goal reviews entered. We also examined choice of eating plan, and whether this was linked to adherence or outcomes. Adherence to the nurse support schedule was assessed by nurse records of the number and format of support sessions delivered. Finally, we examined the feasibility for the main trial of collecting a range of physiological and self-report measures as secondary outcomes. The physiological measures were: waist (in cm, measured midway between the lower rib and iliac crest); height (in cm); fat mass (assessed in kg and %, measured using bioelectrical impedance; Tanita, Japan); blood pressure, measured three times (after 3 minute rest) using clinically validated automated devices (Omron, Japan); fasting lipids (serum cholesterol, HDL, LDL, triglyceride); blood glucose and HbA1c. Self-report measures are described in Table 1.

**Table 1:** Self-report measures evaluated as potential secondary outcomes

**Self-report measures**	**Measure characteristics**
^a^*Socio-demographic questions*	Age, gender, years of education
^a^*Treatment Self-Regulation Questionnaire*[26]	12 items comprising three validated sub-scales assessing three dimensions of motivation to use the POWeR intervention (Controlled-External, Controlled-Introjection and Autonomous-Identification + Integration)
*Food Frequency Questionnaire*	Participants report how often they eat 40 food types in a typical day and week. Developed from a validated measure [27].
*Godin Leisure Time Physical Activity Questionnaire*[28]	4 items assessing time spent on mild, moderate and strenuous activities during the past week
*EQ-5D (Euroqol)*[29]	5 items assessing general health-related quality of life, used to generate Quality Adjusted Life Years for cost-effectiveness analyses.
*Theory of Planned Behaviour questions*	10 items, comprising 2 items assessing each of the Theory of Planned Behaviour [30] constructs (attitude, subjective norms, perceived behavioural control, and intention) completed twice to assess attitudes relating to using the POWeR eating plan and the POWeR physical activity plan
^b^*Therapist perceptions scale*[31]	5 items assessing perceptions of the nurse support provider
^c^*Eating plan acceptability*	7 items asking about the acceptability of the eating plan in terms of satiety, taste, cost and difficulty preparing or obtaining foods
^c^*PETS Adherence Questionnaire*[32]	14 items assessing socially acceptable reasons for non-adherence, followed by 3 items asking how much of the time participants had followed the low calorie eating plan, the low carbohydrate eating plan and the physical activity plan
^d^*Other weight loss support*	8 items asking about use of other interventions during the past year
^d^*Patient Enablement Instrument*[33]	6 items assessing perceived ability to cope with weight problem and general health as a result of health care in the past year.

^a^baseline only; ^b^6 month follow-up only; ^c^6 and 12 month follow-up; ^d^12 month follow-up only.

### Analyses

Descriptive statistics were used to characterise our sample at baseline and to assess completion of intervention components and trial procedures. Where data was missing for calculation of particular statistics we report the number for that analysis. We compared intervention arms at follow-up using ANCOVA for the primary outcome (weight), controlling for baseline values. For comparability with previous trials, the intention to treat analysis of weight changes employed Last Observation Carried Forward to replace missing weight data, i.e. was based on the last recorded weight for each patient (which was baseline or 6 months for those lost to 12 month follow-up). We then calculated mean weight change in each group (with C.I.s) and effect sizes for the difference between the intervention arms and the usual care arm in weight at 12 months, adjusted for baseline weight. Having detected substantial deviations from trial protocol in two practices, these analyses were repeated for the three practices that had followed the protocol correctly.

## Results

### Sample characteristics

**Figure 1:**
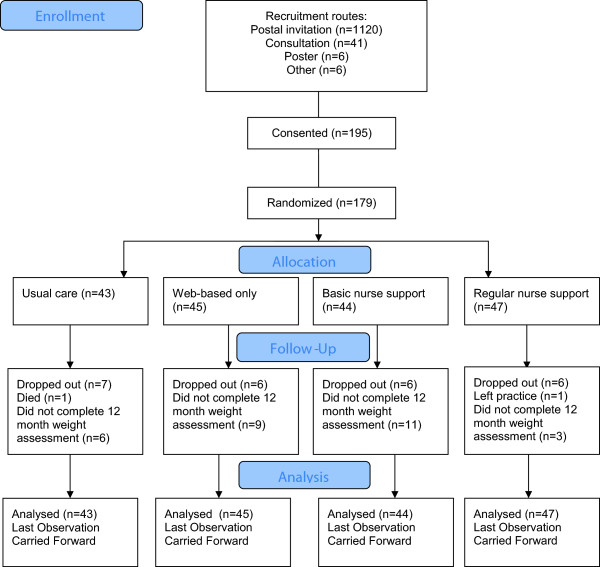
Flow of patients through study.

A total of 179 people were randomised (see Figure 1 for progression through the trial), comprising 15.3% of the total of 1173 people invited to take part. Most participants (n = 132; 73.7%) were recruited by letter, 35 (19.6%) during the consultation, and 12 (6.7%) by poster and other methods. Only 27 participants (15.1%) explicitly discontinued participation in the study, but a further 29 (16.2%) failed to complete the primary outcome (weight) at 12 months, resulting in a total non-completion rate of 31.3%.

Table 2 describes the sample baseline characteristics. Although the majority of participants were female (n = 118; 65.9%) and white (n = 158; 88.3%), over a third of participants were men and the sample included people aged from 20 to 85. Most participants had left education by age 18, and only 36 people (20.1%) reported gaining a university degree. Nearly one in five participants had a BMI of 40 or more (n = 34/178; 19.0%).

**Table 2:** Characteristics of participants at baseline

	**Intervention arm**				**Total sample**
					**(n = 179)**
	**Usual care**	**Web-based only**	**Basic nurse support**	**Regular nurse support**	
	**(n = 43)**	**(n = 45)**	**(n = 44)**	**(n = 47)**	
	**Mean (s.d.)**	**Mean (s.d.)**	**Mean (s.d.)**	**Mean (s.d.)**	**Mean (s.d.)**
Age	49.93 (13.76)	51.16 (13.38)	51.41 (13.02)	52.09 (12.74)	51.17 (13.13)
Age left education (years)	18.47 (4.44)	18.13 (3.40)	17.25 (2.53)	17.98 (2,82)	17.98 (3.34)
BMI	36.18 (4.90)	34.76 (4.44)	36.37 (6.53)	35.35 (5.96)	35.65 (5.52)
Weight (Kg)	103.27 (20.09)	98.25 (18.11)	103.43 (25.23)	100.36 (19.38)	101.28 (20.78)
Male (n, %)	15 (34.9%)	14 (31.1%)	15 (34.1%)	17 (36.2%)	61 (34.1%)

### Receipt of intervention and completion of trial procedures

Website usage was similar across groups (Table 3 shows a mean of around nine weight and goal reviews) but extremely variable among participants, with a range from none to 43 weekly weight and goal reviews completed during the 12 month trial. Of the 136 people randomised to the intervention arms, 19 (14%) never logged onto the website and 32 (23.5%) completed just one or two sessions. However, 46 (33.8%) completed at least nine goal and weight reviews (i.e. 75% of the recommended twelve POWeR sessions); the remaining 39 participants (28.7%) completed between three and eight weight and goal reviews. Of those who chose an eating plan in session 1 (n = 97; 71.3% of the 136 people randomised to the intervention), 69 (71.1%) chose the low calorie plan and 28 (28.9%) chose the low carbohydrate plan.

**Table 3:** Comparison of intervention receipt and weight change in the intervention groups (mean, s.d.; whole sample)

	**Intervention arm**			
	**Usual care**	**Web-based only**	**Basic nurse support**	**Regular nurse support**
	**(n = 43)**	**(n = 45)**	**(n = 44)**	**(n = 47)**
	**Mean**	**Mean**	**Mean**	**Mean**
Mean weight and goal reviews completed	-	8.60 (S.D. 10.43)	7.73 (S.D. 10.75)	9.47 (S.D. 10.37)
Mean sessions of nurse support (all formats)	1.85 (3.48)	1.31 (2.68)	3.11 (2.12)	4.49 (2.82)
Mean sessions of face-to-face nurse support	1.46 (3.08)	1.17 (2.70)	1.80 (1.88)	3.06 (2.75)
Mean sessions of phone nurse support	0.34 (0.94)	0.16 (0.45)	1.00 (1.41)	0.62 (0.97)
Mean sessions of email nurse support	0.05 (0.22)	0.07 (0.33)	0.32 (0.80)	0.81 (1.53)
Weight at 6 months*^a^	99.38 (C.I. 97.66 to 101.10)	99.00 (C.I. 97.32 to 100.68)	97.92 (C.I. 96.20 to 99.63)	97.14 (C.I. 95.50 to 98.78)
Weight at 12 months*^a^	98.89 (C.I. 97.19 to 100.58)	98.92 (C.I. 97.26 to 100.59)	97.02 (C.I. 95.34 to 98.70)	98.76 (97.14 to 100.39)
		(d^b^ = 0.01)	(d = -0.34)	(d = -0.02)
Weight change from baseline at 6 months*	-1.99 (C.I. -0.56 to -3.41)	-2.36 (C.I. -1.17 to -3.54)	-3.45 (C.I. -1.71 to -5.19)	-4.23 (C.I. -1.98 to -6.47)
Weight change from baseline at 12 months*	-2.44 (C.I. -0.85 to -4.03)	-2.30 (C.I. -0.98 to -3.62)	-4.31 (C.I. -2.39 to -6.22)	-2.50 (C.I. -0.63 to -4.38)
Number (%) of participants losing ≥5% of initial body weight at 12 months*	9 (20.9%)	11 (24.4%)	13 (29.5%)	13 (27.7%)

*Intention to treat analysis.

^a^Adjusting for weight at baseline.

^b^d = effect size.

Nurse logs of the support sessions they had offered patients (Table 3) revealed less difference in support levels than intended, with only 1.5 sessions’ difference between the usual care group and regular nurse support group. On investigation, we discovered that two practices had interpreted ‘usual care’ as meaning that they should register the patient on their own nurse-led weight management support programme, rather than continue with the usual care they had been receiving up to that point (i.e. no weight management support). We therefore carried out additional analyses of outcomes in the three practices that had followed protocol by not offering additional nurse support to those in the usual care group (see per protocol analyses below). Even in the per protocol practices, the level of nurse contact was somewhat less than intended, especially in the regular nurse support group, and the level of phone and email contact was very low. There was a skewed distribution of nurse support; in the regular nurse support arm 15/47 (31.91%) patients had no face-to-face support or just one session, a further 15/47 (31.91%) patients had 2 or 3 support sessions, while the remaining 17/47 patients (36.17%) had between 4 and 10 support sessions.

Just under two-thirds of participants attended their practice for follow-up of our primary outcome at 6 months (see Table 4) and only 56.4% completed blood tests. Using our revised follow-up procedures, the follow-up for the primary outcome was increased slightly to 68.7% at 12 months, but the proportion having blood tests dropped to 36.9%. Follow-up rates were highest in the group with regular nurse contact. No serious adverse reactions were reported. Self-report measures were completed at 12 months by a little less than half the sample.

**Table 4:** Completion of trial measures (n, %)

	**Intervention arm**				**Total sample**
					**(n = 179)**
	**Usual care**	**Web-based only**	**Basic nurse support**	**Regular nurse support**	
	**(n = 43)**	**(n = 45)**	**(n = 44)**	**(n = 47)**	
**Objective measures**					
Weight 6 months	24 (55.8%)	28 (62.2%)	25 (56.8%)	36 (76.6%)	113 (63.1%)
Blood test 6 months	22 (51.2%)	24 (53.3%)	23 (52.3%)	32 (68.1%)	101 (56.4%)
Weight 12 months	29 (67.4%)	30 (66.7%)	27 (61.4%)	37 (78.7%)	123 (68.7%)
Blood test 12 months	13 (30.2%)	20 (44.4%)	12 (27.3%)	21 (44.7%)	66 (36.9%)
**Self-report measures (12 months)**					
Quality of Life; EQ-5D	20 (46.5%)	25 (55.6%)	18 (40.6%)	24 (51.1%)	87 (48.6%)
Godin Physical Activity Questionnaire	19 (44.2%)	25 (55.6%)	18 (40.6%)	24 (51.1%)	86 (48.0%)
Food Frequency Questionnaire	18 (41.9%)	24 (53.3%)	17 (38.6%)	20 (42.6%)	79 (44.1%)
Patient Enablement Questionnaire	16 (37.2%)	23 (51.1%)	18 (40.6%)	22 (46.8%)	79 (44.1%)
TPB Questionnaires	N/A	23 (51.1%)	18 (40.6%)	21 (44.7%)	62 (45.6%)
Website Satisfaction Questionnaire	N/A	22 (48.9%)	21 (47.7%)	27 (57.4%)	70 (51.5%)
PETS Adherence Questionnaire	N/A	22 (48.9%)	17 (38.6%)	21 (44.7%)	60 (44.1%)

### Comparison of weight change in intervention arms

At 6 months, weight change was least in the usual care group, only slightly greater in the web-based only group, was substantially increased in the basic nurse support group, and was greatest in the regular nurse support group (see Table 3). However, at 12 months weight change was maintained to a greater degree in the basic nurse support group than the other three groups. The effect sizes for weight at 12 months in the intervention groups compared with usual care, adjusted for baseline weight (ITT analysis), were d = 0.01 for web-based only, d = 0.34 for basic nurse support, and d = 0.02 for regular nurse support. Weight change (based on ITT analysis at 12 months) was very similar among those who selected the low calorie eating plan (mean weight loss = 2.60 kg, s.d. = 5.50) and those who chose the low carbohydrate eating plan (mean weight loss = 2.94 kg, s.d. = 5.31).

The first per protocol analysis we carried out was to repeat all the analyses above excluding the two practices that had deviated from our intended protocol by providing regular nurse support for usual care patients. The pattern of findings was similar to that observed in the whole sample, but with somewhat larger group differences (see Table 5). One third of participants had lost at least 5% of their bodyweight at 12 months in the basic nurse support group, compared with less than half this number in the usual care group. The effect sizes for weight at 12 months in the intervention groups compared with usual care, adjusted for baseline weight (ITT analysis), were d = 0.02 for web-based only, d = 0.55 for basic nurse support, and d = 0.08 for regular nurse support.

**Table 5:** Intervention receipt and weight change in the 3 per protocol practices (mean, s.d.)

	**Intervention arm**			
	**Usual care**	**Web-based only**	**Basic nurse support**	**Regular nurse support**
	**(n = 31)**	**(n = 32)**	**(n = 33)**	**(n = 34)**
	**Mean**	**Mean**	**Mean**	**Mean**
Mean weight and goal reviews completed	-	8.72 (10.45)	9.82 (11.70)	9.82 (10.12)
Mean sessions of nurse support (all formats)	0.59 (1.30)	0.13 (0.43)	2.64 (1.50)	4.06 (2.51)
Mean sessions of face-to-face nurse support	0.45 (1.24)	0	1.39 (1.06)	2.50 (2.14)
Mean sessions of phone nurse support	0.10 (0.31)	0.06 (0.25)	0.82 (1.01)	0.53 (0.71)
Mean sessions of email nurse support	0.03 (0.19)	0.06 (0.36)	0.42 (0.90)	1.03 (1.73)
Weight at 6 months*^a^	97.66 (C.I. 95.59 to 99.73)	97.35 (C.I. 95.31 to 99.40)	95.42 (C.I. 93.42 to 97.43)	94.92 (C.I. 92.94 to 96.90)
Weight at 12 months*^a^	97.57 (C.I. 95.63 to 99.51)	97.48 (C.I. 95.57 to 99.39)	94.63 (C.I. 92.75 to 96.51)	97.13 (C.I. 95.28 to 98.98)
Weight change from baseline at 6 months*	-1.56 (C.I. -0.22 to -2.89)	-1.95 (C.I. -0.76 to -3.14)	-3.16 (C.I. -1.68 to -5.95)	-4.36 (C.I. -1.40 to -7.31)
Weight change from baseline at 12 months*	-1.71 (C.I. -0.31 to -3.10)	-1.76 (C.I. -0.49 to -3.04)	-4.64 (C.I. -2.29 to -6.99)	-2.12 (C.I. 0.22 to -4.46)
Number (%) of participants losing ≥5% of initial body weight at 12 months*	5 (16.1%)	5 (15.6%)	11 (33.3%)	8 (23.5%)

*Intention to treat analysis.

^a^Adjusting for weight at baseline.

The second per protocol analysis we carried out was to examine weight loss in those participants in the intervention groups who completed at least 9 of the recommended 12 sessions and those who did not (pooled across intervention arms to ensure adequate sample sizes as this analysis was carried out only in those providing follow-up weight data). Those who did not complete the POWeR intervention (n = 54) lost an average of 1.50 kg (95% C.I. 0.12 kg to 2.88 kg), whereas those who completed the programme (n = 40) lost an average of 6.70 kg (95% C.I. 4.44 kg to 8.95 kg).

## Discussion

The intention to treat analyses indicated that at 12 months follow-up the usual care, web-based only and regular nurse support groups had lost very similar amounts of weight (2.30 kg to 2.50 kg), whereas the website group with basic nurse support group had lost more weight (4.31 kg). However, one of the most important learning points from this feasibility trial was that practices had interpreted ‘usual care’ differently; two practices had offered patients allocated to usual care an in-practice programme of regular nurse support for weight management (even though this was not routinely offered to obese patients on their practice list), whereas three had followed our planned protocol of providing patients only with their usual medical care. This makes it hard to interpret the comparison with ‘usual care’ in the whole sample, since in three practices we were comparing the POWeR intervention with a no treatment control (as planned), whereas in the two practices that deviated from protocol we were comparing the POWeR intervention with in-person weight management support.

It is more straightforward to interpret the pattern of findings in the three practices that followed protocol. In these practices, at 6 months there was a steady increase in the amount of weight lost with added levels of support; compared with the usual care group the web-based only group lost an additional 0.39 kg on average, the website plus basic nurse support group lost an additional 1.6 kg and the website plus regular nurse support group lost an additional 2.8 kg. Interestingly, at 12 months the web-based only group and website with regular nurse support groups had not sustained these additional gains in weight loss, whereas the website with basic nurse support group had slightly increased their average weight loss to 4.64 kg (compared with 1.71 kg in the usual care group). This pattern of findings was also reflected in the proportion with substantial weight loss (i.e. ≥5% of initial bodyweight); 33.3% of those in the website with basic nurse support group achieved this compared with only 16% of those in the usual care group. Patients who adhered to the intervention (42% of those allocated to one of the website arms) had lost an average of 6.70 kg at 12 months.

These findings are of course only indicative, and require confirmation in our forthcoming fully powered trial, but they do suggest that the combination of the web-based intervention with basic nurse support could provide an effective solution to weight management support in a primary care context. The weight loss achieved in this group was better than the average weight loss achieved by web-based weight management programmes [34] and was very similar to the best of the six face-to-face programmes compared in a UK primary context [35]. Moreover, our sample, although somewhat under-representing men and those from ethnic minorities, was not young or highly educated and can be considered broadly typical of the primary care population eligible to take up this intervention if implemented [35]. However, a significant proportion of patients failed to engage successfully with our web-based intervention, and it is likely that a range of weight management options will be needed for the primary care population [36].

An intriguing finding was that basic nurse support (3 sessions in the first 3 months) actually resulted in better outcomes at 12 months than the more regular support (7 sessions in the first 6 months). This finding was not anticipated, and clearly requires replication as it could simply be due to chance, but it is not inexplicable. Nearly two-thirds of patients in the regular nurse support group attended for less than half the available sessions; it is probable that this group included some patients who were unwilling or unable to access the nurse support, and some may have avoided meeting the nurse if they were failing to lose weight or had lost motivation to adhere to POWeR. However one third of patients in this group did receive regular support for six months. There are indications from the literature that some people given regular support may become dependent on it and find it difficult to maintain motivation independently when it is withdrawn [37], which could explain why the regular nurse support group lost most weight during the period that they had support but then regained it after the support ended. The basic nurse support schedule was timed to provide support during the period when autonomous motivation declines most sharply [38], but may have been too limited to foster dependence, or to become onerous. Another finding of interest was that nearly one in three people chose a low carbohydrate rather than a low calorie eating plan, and outcomes for these two groups were very similar.

This feasibility trial identified some important issues relevant to optimising both the intervention and trial procedures. With regard to adherence to the intervention, patient completion of online sessions appeared comparable to most web-based weight management interventions, in which attrition is typically high [39], and importantly was better than the attrition rates typically observed in interventions with no health professional contact [10,40,41]. Since session completion rates were very similar across all intervention groups, including the web-based only group, this suggests that even the basic contact with primary care involved in nurse recruitment and weight monitoring for the trial at 6 and 12 months may have been sufficient to reduce patient attrition. Findings from a qualitative process study carried out in a sample of participants in this trial support this conclusion (unpublished observation; paper submitted), as several patients in the web-based only group commented that they were motivated by being monitored by their practice nurse. However, implementation of nurse support was lower than intended, with around half the planned support sessions delivered, and this may have diluted the intervention effect. Slightly better outcomes than ours were achieved by a web-delivered intervention when trained coaches provided intensive phone support and succeeded in delivering over 90% of planned support sessions [15]. Support for the POWeR intervention was delivered by practice nurses with a standard clinical workload rather than by dedicated trial staff, hence these follow up rates are likely to represent real world implementation of the intervention; nevertheless, better procedures for maximising delivery of nurse support might improve outcomes in the main trial.

With regard to optimising trial procedures, the main lesson learned was that it is necessary to standardise the intervention to be offered to the control group, rather than relying on practices to continue their ‘usual care’ of recruited patients. Prior to the start of the trial we had established that practices did not routinely offer structured weight loss programmes to their obese patients, but having observed the patient demand for POWeR some practice nurses felt compelled to offer something to patients in the usual care group, and so either improved or developed programmes, drawing partly on their experiences of supporting POWeR. This finding highlights the risk of contamination of the control group in a trial - but also suggests that introducing a programme such as POWeR could raise awareness of the need to offer a range of interventions for helping obese patients. This feasibility trial also highlighted the need to introduce follow-up methods that were more acceptable to patients; while dropout from trials, like intervention attrition, is often high in web-based weight management interventions, some trials have achieved better rates of follow-up than ours. The changes we implemented to our methods of follow-up between the 6 month and 12 month follow-up (as described in the Method) did appear to improve follow-up rates, although we had already permanently lost some patients from the trial at 6 months. This suggests that by allowing patients more flexibility in how and where they are followed up it may be possible to achieve lower rates of dropout.

## Conclusions

This feasibility study provided valuable information about how to optimise and implement a web-based intervention with nurse support in primary care. Our findings identified basic nurse support (3 sessions in 3 months) as the level of support most likely to prove cost-effective, highlighted the need to use a structured intervention as the control arm, indicated that attrition may be reduced by giving patients more choice of how they are followed up, and confirmed that some patients preferred and benefited from a modified low carbohydrate rather than a low calorie eating plan. Most importantly, this study provided an initial demonstration that a combination of web-based and health professional support for weight management could potentially offer a cost-effective means of motivating obese patients in primary care to achieve sustained and clinically meaningful weight loss, and should therefore be further tested in our forthcoming trial.

## Competing interests

No authors have any conflicts of interest to declare. POWeR is a non-commercial web-based intervention developed by the research team using the open source LifeGuide software (http://www.lifeguideonline.org).

## Authors’ contributions

The study was conceived by PL who secured funding with RH, LY, MM, ML, and JP. The design and management of the intervention and trial procedures was carried out by LY, PL, RH, LW, SW, KB, ES, CB, BM and MM. Data analysis was carried out by LY, MM and EA-C. LY produced the first draft of the paper with assistance from ES, with all other authors critically reviewing the content. All authors approved submission.
